# Potentially Deleterious Nonsynonymous Single Nucleotide Polymorphisms (nsSNPs) in the Human *MCPH1* Gene: Systematic In Silico Predictions and Implications for Structure and Function

**DOI:** 10.1155/humu/3241521

**Published:** 2026-09-03

**Authors:** Rizwana Kousar, Sadia Latif, Mohammed Turki Hussain Alharthi, Sidra Tabassum, Muzammil Ahmad Khan, Abdulfatah M. Alayoubi, Morad Qarmali, Muhammad Latif

**Affiliations:** ^1^ Department of Biology, Allama Iqbal Open University, Islamabad, Pakistan, aiou.edu.pk; ^2^ Department of Basic Medical Sciences, College of Medicine, Taibah University, Madinah, Saudi Arabia, taibahu.edu.sa; ^3^ Federal Medical College, Islamabad, Pakistan; ^4^ Gomal Centre of Biochemistry and Biotechnology, Gomal University, Dera Ismail Khan (D.I. Khan), Khyber Pakhtunkhwa, Pakistan, gu.edu.pk; ^5^ Center for Genetics and Inherited Diseases, Taibah University, Madinah, Saudi Arabia, taibahu.edu.sa

**Keywords:** in silico tools, MCPH, *MCPH1*, nsSNPs

## Abstract

**Background:**

The *microcephalin 1*/*BRITI1* (*MCPH1*) gene has been implicated in microcephaly (MCPH), primary ciliary dyskinesia (PCD) syndrome, enlarged cranial volume, and pancreatic and breast cancers. It functions as a tumor suppressor and shows reduced expression in various cancer types. Therefore, the identification of *MCPH1* polymorphisms is of paramount importance.

**Aims and Objectives:**

Considering the multifaceted role of *MCPH1*, this study is aimed at examining the structural and functional impact of nonsynonymous single nucleotide polymorphisms (nsSNPs) on *MCPH1* using in silico tools to understand its potential role in pathogenesis.

**Methodology:**

In the present investigation, a wide array of in silico tools was deployed to explicate putative pathogenic missense/nsSNPs that might impact the structural integrity and molecular functions of *MCPH1*. The mining of damaging nsSNPs was carried out based on the evolutionary conservation of sequence information, prediction of protein stability, interaction analysis, and their potential impact was assessed using structure prediction and 3D modeling, and interaction with other genes/pathways.

**Results:**

In the initial screening, SIFT, PolyPhen‐2, SNAP2, Align GVGD, PhD‐SNP, PANTHER, and SNPs&GO revealed 23 potential deleterious SNPs in *MCPH1*. Population frequency analysis using gnomAD revealed that the prioritized variants were extremely rare in the global population. I‐mutant and ConSurf further revealed that 16 nsSNPs exhibited a decrease in protein stability due to changes in size and charge. I‐TASSER was used to predict wild‐type and mutant protein 3D structures. The potential effect of variation on 3D protein structure was assessed using the Chimera tool, which presented the probable structural loss in eight variants, including p.A11D, p.H49D, p.T59I, p.W60S, p.W60C, p.L74P, p.C79G, and p.L781P. Most of these potential variants resided in the *N*‐terminal BRCT domain, which is implicated in neurodevelopment, and thus may lead to the pathogenesis of primary microcephaly. Furthermore, single‐cell transcriptomic mapping of MCPH1 against dataset of 33,206 cells from malformations of cortical development detected MCPH1 expression across multiple cellular populations. This broad distribution suggested potential MCPH1 function across several neural and glial cell populations.

**Conclusion:**

The potentially deleterious nsSNPs predicted in this study can be of great significance in understanding *MCPH1-*related diseases and may serve as potential targets for future functional validation and therapeutic interventions.

## 1. Introduction

The human genome consists of 3.055 billion base pairs residing in every nucleated body cell [1]. The International 1000 Genome Project Consortium unlocked 4.1–5.0 million sites in which a typical human genome varies from the reference genome, and > 99.9% of the reported variants are single nucleotide polymorphisms (SNPs) [2]. Although multiallelic SNPs do exist, SNPs are usually biallelic and of transversions (A ↔ C or T; and G ↔ C or T) and transition types (A ↔ G or C ↔ T), occurring approximately every 100–300 bp in the human genome [3]. Approximately 50% of SNPs are in the noncoding regions, 25% are synonymous, whereas the remaining 25% are nonsynonymous, accountable for altering amino acid sequence and protein composition thereby affecting protein–protein interactions, protein dynamics, geometry and stability, physicochemical properties, protein folding, charge, hydrophobicity, translation, and posttranslational modification, which may lead to human diseases [4–8].

Bioinformatics tools have been successfully deployed in a substantial number of studies to predict the impact of nsSNPs on the structural integrity and molecular functions of the respective proteins [9–11]. Several studies have revealed the potential role of nsSNPs in various human diseases, such as neurodegenerative diseases, breast cancer, and neurodevelopmental disorders [12–14]. Although the analyses reported in the literature were conducted using in silico tools that necessitate experimental validation, these remain valuable as an initial screening method to minimize labor‐intensive and expensive laboratory procedures [15].

The Human *microcephalin 1*/*BRITI1* (*MCPH1*) gene comprises 14 exons and is located on chromosome 8p23.1 [16]. *MCPH1* encodes a centrosomal protein encompassing three BRCT domains, including *N*‐terminal BRCT1, C‐terminal BRCT2, and BRCT3, and it is mutated in autosomal recessive primary microcephaly (MCPH, OMIM251200) and premature chromosome condensation syndrome [17]. MCPH is characterized by reduced brain size due to defects in neural progenitors during neurodevelopment. Human forebrain organoid models offer a robust framework for understanding the molecular basis of human microcephaly [18]. More than 28 genes that play critical roles in the regulation of neurogenesis have been identified. A total of 39 mutations have been reported in the *MCPH1* gene, including 19 implicated in primary microcephaly (HGMD; accessed on April 18, 2026). *MCPH1* was the first gene identified in a consanguineous family exhibiting a marked reduction in the cerebral cortex [19, 20]. *MCPH1* is downregulated in breast, prostate, and ovarian cancers and acts as a tumor suppressor gene regulated by miR‐27a. *MCPH1* has also been implicated in lung cancer, and its overexpression leads to cell cycle arrest at the S and G2/M phases. *MCPH1* regulates p53 expression via p53 ubiquitination [21]. *MCPH1* is also implicated in aging and age‐related diseases [22].

MCPH is a multifaceted protein that plays a key role in several cellular pathways, including cell cycle progression, DNA damage response, oncogenic pathways, chromatin remodeling, and chromosome condensation [23, 24]. Damaging nsSNPs in *MCPH1* may hamper its function and disturb the underlying molecular pathways. Considering the multifunctional role of MCPH1, the present study was designed to identify potential pathogenic nsSNPs and to elucidate their potential structural and functional impact on the MCPH1 protein using in silico tools. In brief, the mining of damaging nsSNPs was carried out based on the evolutionary conservation of sequence information, prediction of protein stability, 3D structures prediction and modelling, and interaction with other genes/pathways. Filtered predicted pathogenic variants in MCPH1 may serve as a benchmark for understanding disease predisposition and may contribute to the development of future therapeutic strategies following experimental validation.

## 2. Methods

### 2.1. Data Collection

The *MCPH1* sequence was retrieved from the NCBI (https://www.ncbi.nlm.nih.gov) database (accessed on December 04, 2022). The primary sequence of the protein (UniProt accession ID Q8NEM0) was retrieved from UniProt (https://www.uniprot.org/) (accessed on December 12, 2022). SNPs identified in *MCPH1* were obtained from the dbSNP database (https://www.ncbi.nlm.gov/SNP/). Only missense nsSNPs were preferred for further analysis because of their profound effect on the structural arrangement and function of the encoded MCPH1 protein.

### 2.2. Prediction of Deleterious nsSNPs

**Figure 1:**
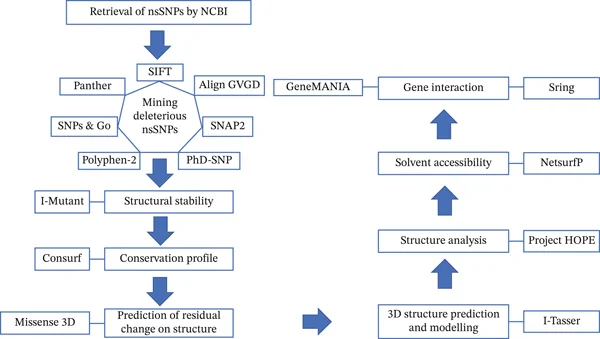
A summary of in silico tools used to retrieve damaging SNPs in the *MCPH1* gene.

Several online tools were integrated to investigate damaging missense nsSNPs reported in MCPH1. A summary of in silico tools used to retrieve damaging SNPs in the *MCPH1* gene is depicted in Figure 1.

Initially, missense nsSNPs of *MCPH1* obtained from the dbSNP database were submitted to Sorting Intolerant from Tolerant (SIFT; https://sift.bii.a-star.edu.sg/; v 5.2.2). SIFT determines the intolerant (probability score < 0.05) and tolerated SNPs (probability score ≥ 0.05) based on sequence alignment and homology. The nsSNPs predicted to be damaging by SIFT were then sequentially submitted to PolyPhen‐2 (Polymorphism Phenotyping v2) (http://genetics.bwh.harvard.edu/pph2/), SNAP2 (https://www.rostlab.org/services/SNAP/) (accessed February 4, 2023), Align GVGD (http://agvgd.hci.utah.edu/) (accessed on March 2, 2023), PhD‐SNP (https://snps.biofold.org/phd-snp/phd-snp.html), and PANTHER 17.0 (http://pantherdb.org/tools/csnpScoreForm.jsp), and SNPs&GO tools (accessed on May 2, 2023) [25–30]. Default parameters were applied to all in silico tools to ensure reproducibility. PolyPhen v2 predicted the amino acid substitution as probably damaging, possibly damaging, or benign, along with a numerical score ranging from 0.0 (benign) to 1.0 (damaging) as output. The output score of SNAP2 ranged from −100 (strong neutral prediction) to +100 (strong effect prediction), which indicated the likelihood of a specific sequence variant to change the native protein function with expected accuracy. In Align GVGD, variants classified as C65 were most likely to affect protein function. PANTHER classified nsSNPs as probably damaging or probably benign based on evolutionary relationship, molecular functions, and their interactions with other proteins.

### 2.3. Analysis of Protein Structural Stability by I‐Mutant

The I‐Mutant‐2 tool (accessed on May 20, 2023) was employed to investigate the effects of single‐point mutations on protein stability. This web server accepts protein sequences or structures as input queries. This support vector machine (SVM)–based tool computes the Gibbs free energy change by analyzing the tertiary structure and sequence. It categorizes mutations as largely unstable (DDG < −0.5 kcal/mol), largely stable (DDG > 0.5 kcal/mol), or neutral (−0.5 ≤ DDG ≤ 0.5 kcal/mol) [31]. Here, the protein sequence of Q8NEM0 and the sequence variant position and mutated residue were used as the input query, and the DDG value and the corresponding increase or decrease in protein stability were used as the output results.

### 2.4. Evolutionary Conservation Analysis of Damaging nsSNPs by ConSurf

The ConSurf web server (http://consurf.tau.ac.il/) (accessed on June 24, 2023) was used to analyze the evolutionary conservation of amino acids in *MCPH1*. The conservation of an amino acid or nucleotide position in a protein molecule depends on its structural and functional significance. The protein sequence in FASTA format was submitted to ConSurf as an input query [32]. Based on the phylogenetic relationship between homologous sequences, this web server computes the conservation score (1–9) of amino acids using a color scheme, thus categorizing amino acids into variable, average, and highly conserved.

### 2.5. Predicting the Effect of Amino Acids on the Structural Integrity of *Microcephalin*

The Missense 3D v.1 (http://missense3d.bc.ac.uk/missense3d/ assessed on July 5, 2024) tool was used to assess the impact of amino acid substitutions on protein structure. The position on protein sequence option was used to input wild‐type amino acids, mutant amino acids, and residue numbers by choosing Q8NEM0 as the Uniprot ID and 3KTf and 3SHT as PDB codes.

### 2.6. 3D Modelling of Wild‐Type Microcephalin and Its Mutants

Nine experimental structures, including 3KTF, 3PA6, 3T1N, 2WT8, 3SHT, 3SZM, 3U3Z, 7C5D, and 3SHV, have been submitted to the PDB (accessed on July 7, 2024). However, none of these covers the full‐length MCPH protein. Therefore, I‐TASSER was used to predict the full‐length structure [33]. The energy minimization of the predicted structures was performed using Chiron and refined using Galaxy [34]. The models with the highest C‐scores were subjected to structural validation using ERRAT, PROCHECK, ProSA‐Web, and SWISS‐MODEL to assess the credibility of the generated models. The SWISS‐MODEL structure assessment tool predicts the MolProbity score and QMEAN Z‐score and approves a model based on a low MolProbity score and a high QMEAN Z‐score. If the ERRAT quality score is > 50%, the model is generally considered to be of good quality [35]. For wild‐type microcephalin, ERRAT computed an overall quality factor of 78.90, whereas the ProSA‐Web and MolProbity SWISS‐MODEL structure assessment tool predicted a 3.11 *Z*‐score, 2.16 MolProbity score, and −9.17 QMEAN *Z*‐score, respectively, indicating that the predicted protein structures satisfied the quality criteria [36]. The negative QMEAN *Z*‐score observed in the generated models indicated instability in the protein structure.

### 2.7. Prediction of *MCPH1* Structure and Mutant Analysis by the Project Have (y) Our Protein Explained (HOPE) Server

HOPE is a web‐based approach for analyzing point mutations in the structural conformation and function of proteins [37]. The protein FASTA sequence is an input query, and the effect of mutation on proteins with text, figures, and animations is the output result.

### 2.8. Solvent Accessibility Prediction by NetsurfP‐2.0

NetSurfP‐2.0 (http://www.cbs.dtu.dk/services/NetSurfP/) server explores the surface accessibility, secondary structure, disorder, and phi/psi dihedral angles of amino acids in an amino acid sequence [38]. The FASTA sequence of the MCPH1 protein was used as the input query, and the buried and exposed regions of the protein structure were obtained as the output.

### 2.9. Gene–Gene Interactions Prediction and Enrichment Analysis

GeneMANIA (http://www.genemania.org/) and Search Tool for Retrieval of Interacting Genes/Proteins (STRING) (http://string-db.org/) (accessed on August 20, 2024) are web‐based tools for assessing and integrating gene–gene interactions. These tools generate information on gene coexpression, colocalization, shared protein domains, and targeted metabolic pathways based on associations with other databases of physical interactions and curated biological pathways [39, 40]. *Microcephalin1* was subjected to GeneMANIA and STRING analyses to generate a gene–gene/protein interaction network.

Furthermore, MCPH1 expression was examined using SCP1815 for enrichment mapping. The gene was queried in the portal′s scatter/UMAP visualization, and expression was displayed using a red intensity scale. Cells were annotated according to the provided ontology labels, including pyramidal neurons, inhibitory interneurons, astrocytes, oligodendrocytes, oligodendrocyte precursor cells, microglia, endothelial cells, and unclassified cells. The analysis was descriptive and based on the portal‐provided normalized expression visualization.

## 3. Results

### 3.1. SNPs Recruitment

**Figure 2:**
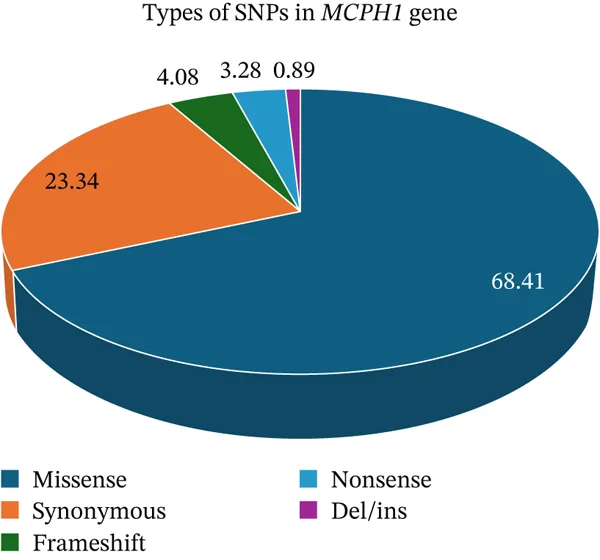
Distribution of SNPs in different functional classes of the *MCPH1* gene.

SNPs reported in the human *MCPH1* gene were obtained from the NCBI database (https://www.ncbi.nlm.nih.gov/SNP/snp_ref.cgi?locusId=79648). It comprised a total of 1127 SNPs, of which 771 were categorized as missense (nsSNP), 263 as synonymous, 46 as frameshift, 37 as nonsense, and 10 as deletion/insertions (Figure 2). The present study was performed on nsSNPs of the *MCPH1* gene only, which accounted for 68.5% of the total SNPs reported in the human *MCPH1* gene.

### 3.2. Screening of Deleterious nsSNPs in *MCPH1*

Missense SNPs associated with the *MCPH1* gene were subjected to in silico analysis using various bioinformatics tools, including SIFT, Polyphen‐2, SNAP2, Align GVGD, PhD‐SNP, PANTHER, and SNPs&GO. For the initial screening of deleterious SNPs, SIFT predictions were performed for 771 missense SNPs. Among these, SIFT classified 331 nsSNPs as damaging. To filter the SIFT results, several other tools, including Polyphen‐2, SNAP2, Align GVGD, PhD‐SNP, PANTHER, and SNPs&GO, were used. The proportion of damaging variants predicted by these tools included 278 by Polyphen‐2, 289 by SNAP2, 184 by Align GVGD, 75 by PhD‐SNPs, 160 by Panther, and 34 by SNPs&GO. Among all these variants, 23 were considered the most deleterious (Table 1). These variants were considered for further analysis because of their potential pathogenicity.

**Table 1:** List of deleterious SNPs predicted in the *MCPH1* gene by deploying seven prediction tools.

S. no.	rs# SNP id	Chro. position	SNP	Position	AA change	SIFT		Polyphen		SNAP2		Align GVGD		PhD‐SNP		Panther			SNPs&GO	
						Pred	Pro	Pred	Pro	Pred	Score	Class	GD score	Pred	Score	Pred	Preservation time	Score	Pred	Score
1	rs767448816	6409288	C/A	11	A/D	D	0	ProD	1	E	21	C65	99.13	D	8	ProD	455	0.57	D	0
2	rs372088330	6414795	C/G	49	H/D	D	0	ProD	1	E	92	C65	81.24	D	3	ProD	1368	0.86	D	1
3	rs1333885096	6414826	C/G	59	T/I	D	0	ProD	1	E	51	C65	89.28	D	5	ProD	750	0.74	D	1
4	rs751733149	6414829	G/C	60	W/S	D	0	ProD	1	E	93	C65	176.5	D	1	ProD	750	0.74	D	1
5	rs1240826704	6414830	G/C	60	W/C	D	0	ProD	1	E	78	C65	214.3	D	3	ProD	750	0.74	D	0
6	rs1278624882	6414871	T/C	74	L/P	D	0	ProD	1	E	63	C65	97.78	D	7	ProD	1368	0.86	D	1
7	rs1349038447	6431500	T/G	79	C/G	D	0	ProD	1	E	78	C65	158.23	D	7	ProD	1628	0.89	D	3
8	rs772186443	6436054	T/C	110	C/R	D	0	ProD	1	E	77	C65	179.5	D	3	ProD	750	0.74	D	0
9	rs368136293	6436055	G/A	110	C/Y	D	0	ProD	1	E	69	C65	193.7	D	3	ProD	750	0.74	D	0
10	rs376996626	6480817	C/T	693	R/C	D	0	ProD	1	E	69	C65	179.5	D	7	ProD	1368	0.86	D	0
11	rs761698331	6480847	C/T	703	R/C	D	0	ProD	1	E	58	C65	179.5	D	8	Pos D	324	0.5	D	5
12	rs988591006	6480854	G/A	705	C/Y	D	0	ProD	1	E	60	C65	193.7	D	8	ProD	1368	0.86	D	7
13	rs1366813272	6480857	G/C	706	W/S	D	0	ProD	1	E	76	C65	176.5	D	9	ProD	1368	0.86	D	8
14	rs1344916776	6480869	A/G	710	Y/C	D	0.01	ProD	1	E	45	C65	193.7	D	4	Pos D	324	0.5	D	2
15	rs753362195	6480874	T/G	712	W/G	D	0	ProD	1	E	79	C65	183.7	D	7	ProD	1628	0.89	D	4
16	rs758843237	6480875	G/C	712	W/S	D	0	ProD	1	E	75	C65	176.5	D	8	ProD	1628	0.89	D	6
17	rs778229284	6480876	G/C	712	W/C	D	0	ProD	1	E	56	C65	214.3	D	8	ProD	1628	0.89	D	6
18	rs746294778	6499874	G/T	720	G/V	D	0	ProD	0.99	E	33	C65	108.7	D	5	ProD	144	0.57	D	1
19	rs758637845	6621503	T/C	755	L/P	D	0	ProD	1	E	76	C65	97.79	D	6	ProD	1368	0.86	D	3
20	rs1208842915	6621581	T/C	781	L/P	D	0	ProD	1	E	76	C65	97.78	D	7	ProD	1368	0.86	D	3
21	rs189716626	6621586	G/A	783	G/R	D	0	ProD	1	E	77	C65	125.1	D	1	ProD	1628	O.89	D	0
22	rs781673106	6621617	C/A	793	A/D	D	0	ProD	1	E	77	C65	125.7	D	6	ProD	1368	0.86	D	2
23	rs777703312	6621691	G/A	818	D/H	D	0	ProD	1	E	73	C65	81.24	D	1	ProD	1036	0.85	D	0

Abbreviations: AA, amino acid; Chr, chromosome; D, damaging; E, effect; Pos D, possibly damaging;Pred, prediction; Prob, probability; ProD, probably damaging.

### 3.3. Variant Frequency

The gnomAD (https://gnomad.broadinstitute.org/ assessed on April 15, 2026) was used to evaluate the frequency of commonly predicted nsSNPs. The findings revealed a very low frequency (< 0.0001) in the global population database, which supports the findings of the prediction tools deployed in the current study.

### 3.4. Prediction of MCPH1 Protein Structural Stability

Among the 23 variants, 21 (p.A11D, p.H49D, p.T59I, p.W60S, p.W60C, p.L74P, p.C79G, p.C110R, p.R693C, p.R703C, p.C705Y, p.W706S, p.Y710C, p.W712G, p.W712S, p.W712C, p.G720V, p.L755P, p.L781P, p.G783R, and p.D818H) were predicted to potentially decrease protein stability by I‐Mutant‐2 (Table S1) due to changes in the charge, size, and hydrophobicity of the alternate variant, thus resulting in potential conformational strain.

### 3.5. Amino Acid Conservation Profile of *MCPH1* Protien

**Figure 3:**
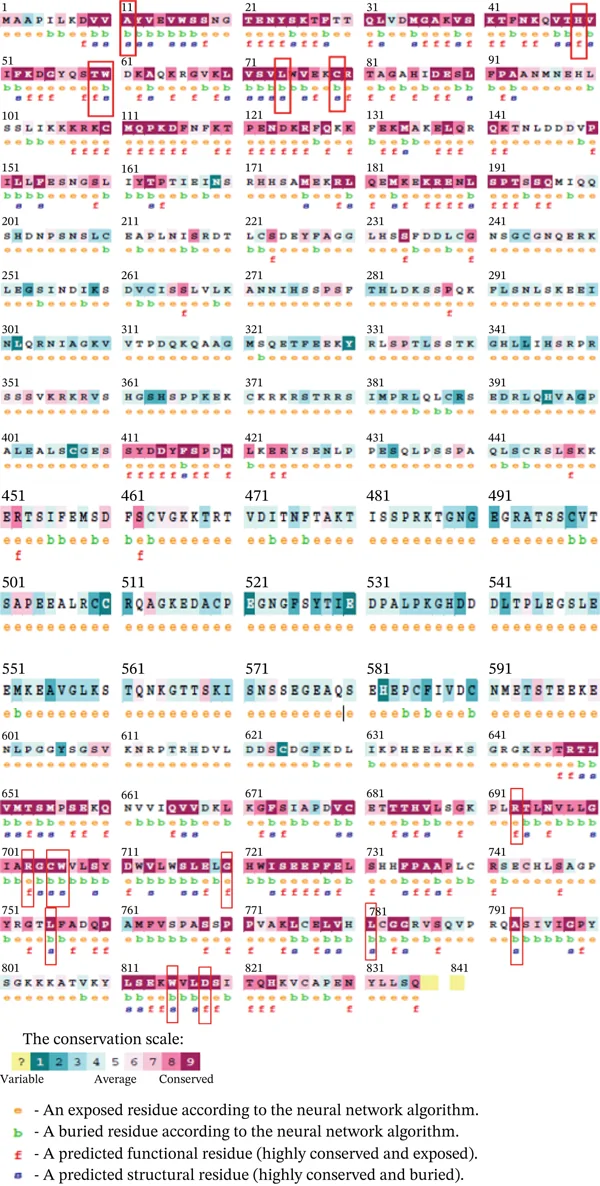
Conservation profile of *MCPH1* protein (amino acids 1–841) using ConSurf.

The ConSurf web server was used to analyze 23 nsSNPs to assess evolutionary conservation for putative structural and functional impact. The ConSurf output predicted 16 variants (p.A11D, p.H49D, p.T59I, p.W60S, p.W60C, p.L74P, p.C79G, p.R693C, p.R703C, p.C705Y, p.W706S, p.G720V, p.L755P, p.L781P, p.A793D, and p.D818H) as highly conserved residues (conservation score of 9). Among these highly conserved residues, six variants were predicted to be functional and exposed, whereas the remaining 10 variants were of structural significance and were found to be buried (Figure 3 and Table S1).

The Missense3D tool was deployed to predict the impact of 16 potential variants on the *MCPH1* structure following amino acid substitutions. The Missense3D tool detected potential structural damage in eight mutants: p.A11D, p.H49D, p.T59I, p.W60S, p.W60C, p.L74P, p.C79G, and p.L781P. Briefly, eight missense variants showing consistent findings of deleteriousness using in silico pathogenicity prediction tools, depicting potential protein structural and evolutionary consequences and demonstrating low population allele frequency were prioritized. Two prioritized variants (rs372088330 and rs1278624882) were reported in ClinVar with uncertain significance (Table S2). For the remaining variants, no ClinVar and/or HGMD records were available at the time of analysis (accessed on August 1, 2026). The absence of ClinVar records reflected the lack of submitted or curated clinical evidence rather than evidence of benignity. Consequently, these variants were further investigated using additional in silico structural and functional analyses.

### 3.6. Microcephalin 3D Full‐Length Structure Prediction and 3D Modelling

Wild‐type *microcephalin* and the mutants were submitted to I‐TASSER, which is a comparatively advanced tool that recognizes structural templates from the PDB using a multiple threading approach. I‐TASSER predicted five models for the understudied full‐length proteins comprising 835 amino acids and each of its mutants. The generated structures were submitted to Chiron for energy minimization (Table 2) and were further refined using GalaxyWeb. The eight mutant predicted models were validated using validation tools, and the results showed that all the generated mutant models were of good quality (Table 3).

**Table 2:** Initial and final numbers of clashes predicted by the Chiron.

S. no.	SNP ID	Structure	Initial number of clashes	Final number of clashes
		Wild type	488	268
1	rs767448816	p.A11D	836	329
2	rs372088330	p.H49D	828	434
3	rs1333885096	p.T59I	838	431
4	rs1240826704	p.W60C	830	348
5	rs751733149	p.W60S	556	317
6	rs1278624882	p.L74P	756	330
7	rs1349038447	p.C79G	775	423
8	rs1208842915	p.L781P	783	303

**Table 3:** Score prediction using ERRAT, ProSA‐web, and the SWISS‐MODEL structure assessment tool for generated models.

S. no.	SNP ID	Structure	ERRAT	ProSA‐web Z‐score	SWISS‐MODEL structure assessment tool	
—	—	—	—	—	MolProbity score	QMEAN *Z* score
—	—	Wild type	78.90	−3.11	2.16	−9.17
1	rs767448816	p.A11D	75.18	−3.88	2.14	−6.54
2	rs372088330	p.H49D	79.5	−3.37	2.41	−7.59
3	rs1333885096	p.T59I	71.92	−4.02	2.19	−6.91
4	rs1240826704	p.W60C	64.87	−4.67	2.27	−6.67
5	rs751733149	p.W60S	66.20	−4.67	2.27	−6.67
6	rs1278624882	p.L74P	75.62	−3.82	2.30	−7.19
7	rs1349038447	p.C79G	79.5	−3.26	2.48	−7.98
8	rs1208842915	p.L781P	70.46	−3.60	2.04	−6.91

The predicted models for mutants with the highest C values were superimposed on the wild‐type model using TM‐Align, yielding TM‐score and RMSD values (Table 4).

**Table 4:** TM align predicted the TM score and RMSD values for the eight MCPH1 mutants.

S. no.	SNPs ID	Mutant	TM score	RMSD value
1	rs767448816	p.A11D	0.218	8.60
2	rs372088330	p.H49D	0.215	8.68
3	rs1333885096	p.T59I	0.218	8.73
4	rs1240826704	p.W60S	0.177	8.88
5	rs751733149	p.W60C	0.215	8.48
6	rs1278624882	p.L74P	0.219	8.72
7	rs1349038447	p.C79G	0.216	8.62
8	rs1208842915	p.L781P	0.218	8.50

**Figure 4:**
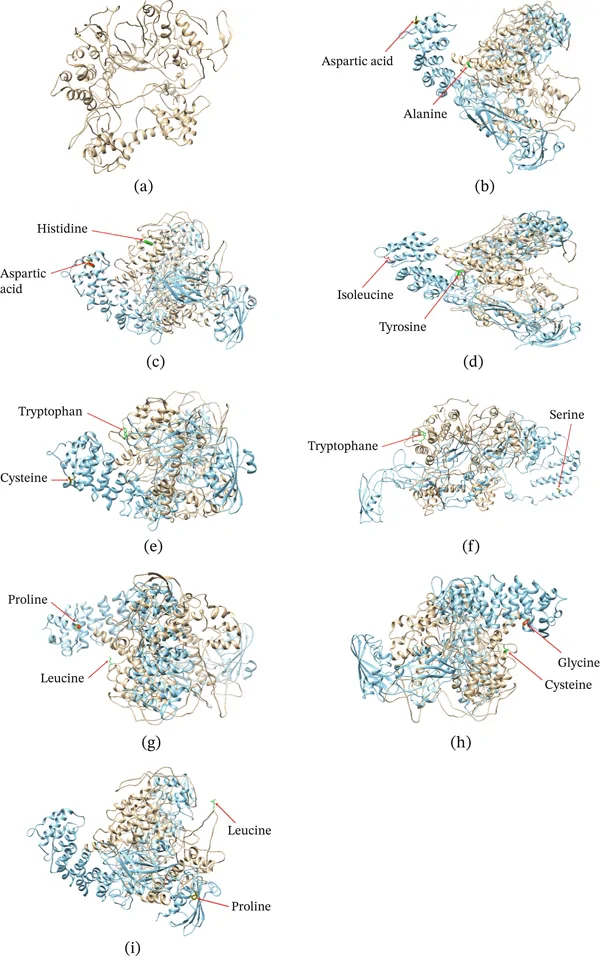
(a) Structure of the wild‐type MCPH1 protein. (b) Superimposed wild‐type MCPH1 protein and mutant having an alanine‐to‐aspartic acid mutation at Position 11 (A11D). (c) Superimposed wild‐type MCPH1 protein and mutant having a histidine‐to‐aspartic acid mutation at Position 49 (H49D). (d) Superimposed wild‐type MCPH1 protein and mutant having a tyrosine‐to‐isoleucine mutation at Position 59 (T59I). (e) Superimposed wild‐type MCPH1 protein and mutant having a tryptophan‐to‐cysteine mutation at Position 60 (W60C). (f) Superimposed wild‐type MCPH1 protein and mutant having a tryptophan‐to‐serine mutation at Position 60 (W60S). (g) Superimposed wild‐type MCPH1 protein and mutant having a leucine‐to‐proline mutation at Position 74 (L74P). (h) Superimposed wild‐type MCPH1 protein and mutant having a cysteine‐to‐lycine mutation at Position 79 (C79G). (i) Superimposed wild‐type MCPH1 protein and mutant with a leucine‐to‐proline mutation at Position 781 (L781P).

UCSF Chimera 1.16 was used to superimpose predicted mutant structures showing the highest RMSD values over the predicted wild‐type microcephalin (Figures 4a–i). The topological similarity between the predicted wild‐type and mutant proteins was assessed using the TM score, whereas the mean distance between the backbone atoms of both proteins was depicted by RMSD values. The TM‐score provides information about the topological similarities between two proteins, and RMSD values show the average distance between the backbone atoms of the wild‐type and mutant proteins. High RMSD values of the mutant structures indicated a greater deviation from the wild type.

### 3.7. Analysis for Mutation Effects on the Structure and Function of the MCPH1 Protein From Project HOPE

**Figure 5:**
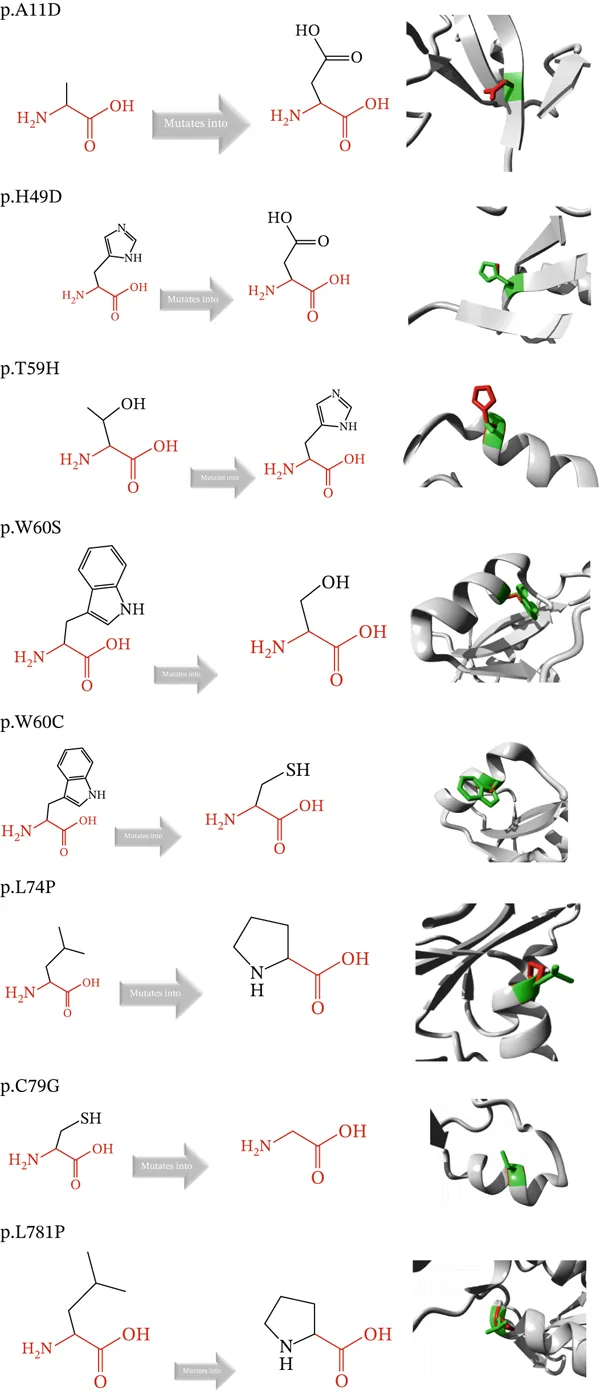
Structural changes predicted by project HOPE for the MCPH1 mutant protein. The protein is colored gray. The side chains of both the wild‐type and mutant residues are shown in green and red, respectively.

Project HOPE software was used to obtain information regarding the potential effect of mutant residues on the structure and function of proteins based on size, hydrophobicity, and charge. HOPE predictions demonstrated differences in the properties of the wild‐type and mutant residues and the possible effects of polymorphic amino acids on the domain and conservation (Table 5). 3D predictions of the MCPH1 protein are illustrated as a comparison of wild‐type and mutant residues (Figure 5).

**Table 5:** HOPE server predicted the effect of residual change on MCPH1 structure and function.

	Amino acid properties	Location of amino acid	Effect of polymorphism on protein structure and function
p.A11D	There is a difference in charge, size, and hydrophobicity between the wild‐type and mutant amino acids.	The mutation is located within BRCT1 domain, and the residue is found buried in the core of the domain.	The mutant residue introduced a charge and was larger than the wild type. It probably will not fit and result in the loss of hydrophobic interactions, which can lead to protein folding problems.
p.H49D	There is a difference in charge and size between wild‐type and mutant amino acids.	The mutation is located within the BRCT1 domain. The residue is found buried in the core of the domain.	The mutant residue can lead to protein folding effects due to its negative charge. Additionally, it is predicted to cause space in the core of the protein due to its smaller size, which can disturb this domain and abolish its function
p.T59H	The wild‐type and mutant amino acids differed in size and hydrophobicity.	The mutation is located within BRCT1 domain. The residue is found buried in the core of the domain.	The mutant residue is larger and will probably not fit, causing the loss of hydrophobic interactions in the core of the protein.
p.W60S	The wild‐type and mutant amino acids differed in size and hydrophobicity.	The mutation is located within BRCT1 domain The residue is buried in the core of the domain.	The mutant residue lost hydrogen bonding with glutamic acid at Position 14. Due to the smaller size, the mutant residue may cause an empty space in the core of the protein and might disturb the core structure of this domain.
p.W60C	The mutant residue was smaller than the wild‐type residue.	The mutation is located within BRCT1 domain The residue is buried in the core of the domain.	The mutation may cause an empty space in the core of the protein and has potential impact on protein folding.
p.L74P	The mutant residue was smaller than the wild‐type residue.	The mutated residue is located on the surface of the BRAT1 domain, with an unknown function.	Due to the size difference, the mutation is predicted to result in loss of external interactions.
p.C79G	The wild‐type and mutant amino acids differed in size and hydrophobicity.	The residue is buried in the core of the BRCT1 domain.	This mutation introduces a glycine at this position. Glycine is highly flexible and can disrupt the required rigidity of the protein at this position.
p.L781P	The mutant residue was smaller than the wild‐type residue.	The mutation is buried in the core of the BRCT3 domain.	The mutation may induce an empty space in the core of the protein thus potential disturbance in the core structure of this domain.

### 3.8. Prediction of Solvent Accessibility

**Figure 6:**
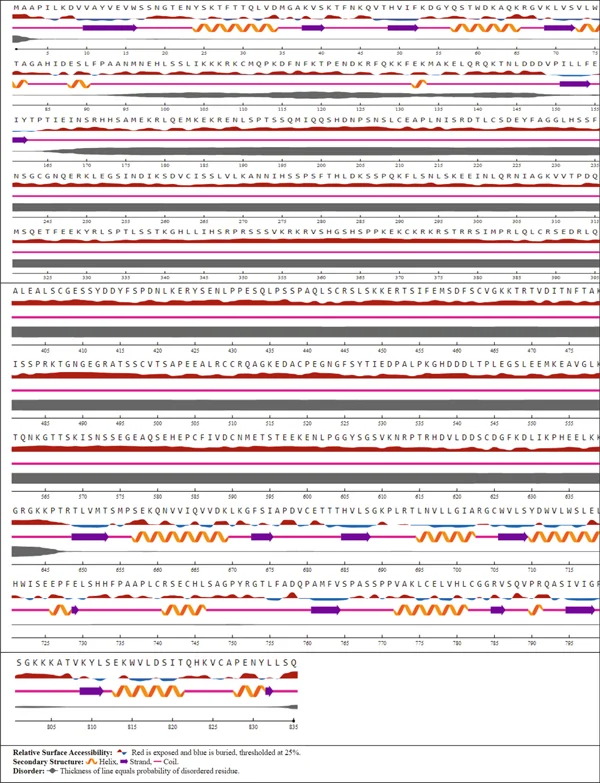
Surface accessibility and secondary structure of *MCPH1* protein.

NetsurfP was used to investigate the potential solvent accessibility and stability of eight nsSNPs (p.A11D, p.H49D, p.T59I, p.W60S, p.W60C, p.L74P, p.C79G, and p.L781P). Among these potentially damaging nsSNPs, the p.L74P variant and its respective wild type were found on the surface, whereas six variants (p.A11D, p.H49D, p.T59I, p.W60S, p.W60C, and p.C79G) and their respective wild variants were found buried (Figure 6 and Table S3). For one variant, NetSurfP did not provide any prediction.

### 3.9. Analysis of the *MCPH1* Gene and Protein Interaction With Other Genes and Proteins

**Figure 7:**
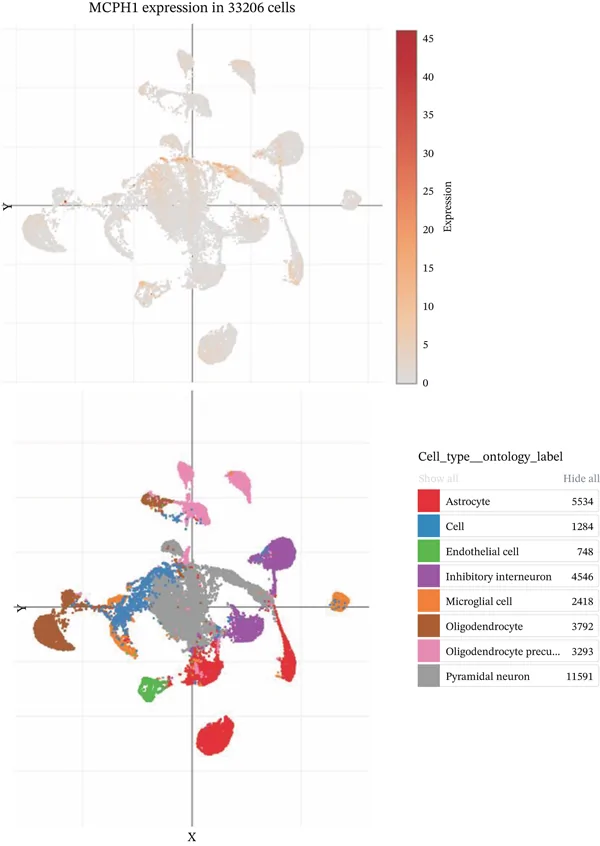
Heterogeneous MCPH1 expression across neuronal and glial cell populations in cortical malformations.

Allelic variants of a gene interact with other genetic and environmental factors, thus increasing or decreasing the susceptibility to diseases [41]. GeneMANIA predicted a composite gene–gene interaction network, in which *MCPH1* showed functional potential interactions with 20 other genes. These genes included *NCPAH2*, *NCPAG2*, *NCAPD3*, *SET*, *CRACR2A*, *SMC4*, *SMC2*, *BRCA1*, *TERF2IP*, *E2F2*, *TERF2*, *PAXIP1*, *PES1*, *TOPBP1*, *E2F1*, *NCCRP1*, *CCDC77*, *B3GALT6*, *COL27A1*, and *C6orf62*. The top 20 genes predicted by STRING were *TERF2*, *CHD4*, *ATM*, *E2F1*, *BRCA2*, *FOXP2*, *H2AFX*, *RBBP8*, *STIL*, *CENPJ*, *WDR62*, *CEP152*, *ASPM*, *PCNT*, *CDK5RAP2*, *CEP135*, *NCAPD3*, *SET*, *NCAPG2*, and *CDC27* (Figure S1). *ASPM*, *WDR62*, *STIL*, *CENPJ*, *CEP152*, *PCNT*, *CDK5RAP2*, and *CEP135* have been implicated in the proliferation of neural progenitors, whereas *BRCA1*, *ATM*, *E2F1*, *TOPBP1*, *H2AFX*, and *RBBP8* have profound contributions to the cell cycle and DNA damage pathways. Thus, mutated *MCPH1* variants potentially impair the normal functioning of interacting proteins, leading to disease. Furthermore, Single‐cell transcriptomic mapping of MCPH1 was performed in a dataset of 33,206 cells, with pyramidal neurons representing the largest population (11,591 cells), followed by astrocytes (5534 cells) and inhibitory interneurons (4546 cells). MCPH1 expression was distributed across the UMAP rather than concentrated within one distinct cluster. Most cells showed weak expression, whereas scattered cells and localized regions exhibited relatively higher signal (Figure 7). MCPH1‐positive cells were observed within neuronal and nonneuronal compartments, indicating heterogeneous expression across the cortical cell landscape.

## 4. Discussion

SNPs are variations in the DNA sequence that occur with a frequency of ≥ 1% in the human population and are considered the most abundant form of genetic variation. SNPs are crucial in disease prognosis and serve as biomarkers for predicting the body′s response to various treatment regimens in pharmacokinetics, thereby influencing the field of precision medicine. Several in silico studies have been performed to predict whether nsSNPs have deleterious effects on the encoded proteins [9–11, 42]. However, the prediction precision depends on the authentic and accurate input of raw data. Therefore, it is recommended to use multiple tools for a single task and compare the outputs to develop a consensus. Furthermore, in silico results should be validated using gold‐standard wet‐lab experiments.

The *MCPH1* gene encodes a centrosomal protein with three BRCT (*BRCA1* C‐terminal) domains that are mutated in autosomal recessive primary microcephaly and premature chromosome condensation syndromes [17]. *Microcephalin1* is downregulated in various cancer types, including breast, prostate, and ovarian cancers, and acts as a tumor suppressor gene [43]. Several missense and nonsense mutations in the microcephalin gene have been attributed to MCPH [23, 44]. The association of MCPH1 polymorphisms has been established in various diseases, including bipolar disorder and schizophrenia [45], breast cancer [46], larger cranial volume [47], late‐onset Alzheimer′s disease [48], and impaired neurocognitive function in patients with glioma.

In the present study, a total of 1127 SNPs were obtained from dbSNP, of which 771 nonsynonymous were subjected to seven in silico tools, including SIFT, Polyphen‐2, SNAP2, Align GVGD, PhD‐SNP, PANTHER, and SNPs&GO. These bioinformatics tools predicted 23 nsSNPs to be potentially deleterious, which were further subjected to analysis to investigate their potential damaging impact on the encoded protein. Prioritized variants were very rare (MAF < 0.0001). Furthermore, 50% of the prioritized potential eight variants depicted a Combined Annotation‐Dependent Depletion (CADD) score ≥ 20, whereas the rest depicted a score > 30, predicted to be among the 1% and 0.1% most pathogenic variants. Based on the ensemble predictor, Rare Exome Variant Ensemble Learner (REVEL), all variants exhibited REVEL scores > 0.5, except one, suggesting a higher probability of deleterious impact on the MCPH1 protein.

All prioritized potentially pathogenic SNPs decreased protein stability, as predicted by the I‐Mutant‐2, except for C110Y and A793D. Less stable proteins are degraded, misfolded, or aggregated, resulting in cellular dysfunction [49]. The evolutionary conservation profile of a protein accounts for the severity of detrimental mutations. Missense SNPs residing in highly conserved regions are more likely to be detrimental than missense SNPs found in variable regions [50]. ConSurf analysis of MCPH1 conservation predicted 10 variants, including p.A11D, p.W60S, p.W60C, p.L74P, p.C79G, p.R703C, p.C705Y, p.W706S, p.L755P, and p.L781P as highly conserved and buried residues, whereas six variants, including p.H49D, p.T59I, p.R693C, p.G720V, and p.D818H, were predicted to be highly conserved and exposed.

A missense 3D tool was used to assess the potential effects of 16 nsSNPs on the structural stability of *MCPH1*. Eight nsSNPs were predicted to cause 3D structural damage. Hence, these eight nsSNPs were selected for further in silico analyses. I‐TASSER was used to predict the structure of *MCPH1* and its mutants. Based on the analysis using Chimera, potentially damaging SNPs were predicted to have detrimental effects on protein structure and, hence, may compromise its function. In the current study, increased RMSD values were observed for the predicted structures of mutant *MCPH1* compared with the wild‐type, predicting that the variants may hamper the structural integrity of the protein.

The HOPE project predicted the impact of nsSNPs on protein domains and structures by considering the physicochemical properties of wild‐type and mutated residues. It was observed that seven of the potentially damaging mutations predicted in the present study were located in the BRCT1 domain, and one is in the BRCT3 domain. Predicted mutations can alter the interaction of the *N*‐terminal BRCT domain with the chromatin remodeling complex SWI/SNF and may disturb DNA repair mechanisms [51]. Likewise, predicted mutations can interrupt ionizing radiation (IR)–induced foci (IRIF) formation and E2F1‐mediated transcriptional regulation of Chk1 and BRCA1 at the G2/M checkpoint [52, 53], which may result in disease development. The *N*‐terminal BRCT domain is also implicated in brain development. A mouse model lacking the N ^′^‐BRCT domain of *MCPH1* exhibited reduced brain size [48]. Most of the loss‐of‐function mutations reported in *MCPH1* are located in Exons 2 and 3, encoding the *N*‐terminal BRCT domain, providing insight into domain‐specific vulnerability and its relevance to neurodevelopment. The solvent‐accessible surface area of a protein classifies the amino acid residues as potentially exposed or buried and affects protein folding and stability [54, 55]. Solvent accessibility analysis using NetsurfP predicted that one variant (L74P) and its respective wild type were exposed to the surface, whereas six variants (p.A11D, p.H49D, p.T59I, p.W60S, p.W60C, and p.C79G) and their respective wild‐type variants were found to be potentially buried. Mutations in buried residues may lead to profound effects on structural integrity, whereas mutations in exposed residues result in functional consequences.

Gene–gene interaction analysis was performed to investigate the interacting potential partners of the MCPH1 protein. The damaging nsSNPs predicted in the present study may affect the interacting network of *MCPH1* with other proteins, especially *ASPM*, *STIL*, *CEP135*, *CDK5RAP2*, *WDR62*, *SMC4*, *BRCA1*, *and BRCA2*, and thus may hamper relevant cellular pathways. Single‐cell transcriptome mapping of MCPH1 performed against a dataset of 33,206 cells revealed its diffused and heterogeneous expression pattern consistent with a role that is not exclusive to one cortical cell lineage. Expression across neuronal, glial, and progenitor‐associated populations indicated potential influence of altered MCPH1 function on multiple developmental processes relevant to cortical malformations.

The limitations of the present study include predictive findings rather than definitive as these are based entirely on computational analyses. Although several in silico tools were deployed to prioritize variants and evaluate their potential impact on protein structure, stability, evolutionary conservation, and structure, computational predictions cannot establish pathogenicity. In addition, structural models of potential mutant proteins were generated through computational modeling representing predicted conformations rather than experimentally determined structures. Consequently, the observed structural alterations should be interpreted as speculated rather than confirmed molecular changes. Furthermore, none of the disease models were considered for performing the current in silico analysis. Alleles retrieved from databases physically exist in the population. However, to demonstrate autosomal recessive disorders such as MCPH, both alleles must be inherited following a homozygous recessive pattern. The presence of both recessive alleles in a single individual can be either by chance or due to consanguineous marriage. Hence, regardless of the inheritance pattern, the potential damaging effects of nsSNPs have been hypothesized. SNPs found in the noncoding regions of the *MCPH* gene were not included in the present investigation, despite their profound impact on gene expression.

## 5. Conclusion

The current study predicted the potential impact of nsSNPs on the structural integrity that may impair the function of the MCPH1 protein. MCPH1 is a multifaceted protein that plays a crucial role in various cellular pathways, including cell cycle progression, DNA damage signaling and repair, chromosome condensation, brain development, and tumorigenesis. *MCPH1* is comprised of three BRCT domains that mediate protein–protein interactions with its partners. For instance, BRCT domains interact with phosphorylated proteins during DNA damage and repair signaling. Thus, the structural conformation of BRCT domains is essential for executing these vital functions. All eight nsSNPs prioritized in the current study lie in the BRCT domains and lead to a potential decrease in protein stability. Based on computational tools, the present study provides insights into the potentially damaging impact of nsSNPs that might hamper protein structure and function. Filtered potential pathogenic disease variants in *MCPH1* may be a benchmark for understanding disease predisposition and will help in designing future therapeutic interventions following experimental validation. The present study may assist molecular geneticists in interpreting variant pathogenicity and designing therapeutic interventions required for personalized medicine. However, additional in vitro and in vivo experimental and clinical studies are required to validate prioritized candidate variants.

## Author Contributions

Conceptualization: R.K. and M.A.K. Methodology: S.L., S.T., R.K., M.Q., and M.L. Data collection: S.L., A.M.A., S.T., M.T.H.A., and M.A.K. Formal analysis: M.Q., S.T., R.K., and M.Q. Software: R.K., S.T., S.L., M.Q., and M.T.H.A. Resources: R.K., M.A.K., and M.L. Writing—original draft preparation: R.K., M.Q., S.L., S.T., M.A.K., A.M.A., M.T.H.A., and M.L. Writing—review and editing: S.L, R.K., S.T., M.A.K., M.T.H.A., M.Q., A.M.A., and M.L. Visualization: A.M.A., R.K., S.S., M.T.H.A., and M.L. Supervision: M.A.K. and R.K. Project administration: R.K., M.A.K., and M.L. Funding acquisition: R.K.

## Funding

This project was funded by the Research, Development and Innovation Authority, Kingdom of Saudi Arabia, Award Number (12996‐iau‐2023‐TAU‐R‐3‐1‐HW‐).

## Disclosure

All authors have read and agreed to the published version of this manuscript.

## Ethics Statement

This study did not require ethical approval. No animals were involved in this study.

## Consent

The authors have nothing to report.

## Conflicts of Interest

The authors declare no conflicts of interest.

## Supporting information


**Supporting Information** Additional supporting information can be found online in the Supporting Information section. Table S1: Prediction of structural stability and evolutionary conservation for selected deleterious SNPs in *MCPH1*. Table S2: A summary of available ClinVar annotations for the prioritized missense variants. Table S3: Solvent accessibility and stability profile of *MCPH1*. Figure S1: The interaction network of *MCPH1* with other genes: (a) GeneMANIA prediction and (b) STRING prediction.

## Data Availability

All data generated or analyzed during this study are included in this article and its Supporting Information files.
