# Supplementary material for: Potentially Deleterious Nonsynonymous Single Nucleotide Polymorphisms (nsSNPs) in the Human MCPH1 Gene: Systematic In Silico Predictions and Implications for Structure and Function
Source: Hum Mutat. 2026 Sep 3;2026:3241521. doi: 10.1155/humu/3241521 (PMC13539433; doi:10.1155/humu/3241521)
Supplement: Supplementary file 1 [file HUMU-2026-3241521-s001.pdf]

Potentially Deleterious Non-Synonymous Single Nucleotide Polymorphisms (ns SNPs) in the Human MCPH1 Gene: Systematic *In silico* Predictions, Implications for Structure and Function

Rizwana Kousar[1, *], Sadia Latif[1], Mohammed Turki Hussain Alharthi[2], Sidra Tabassum[3], Muzammil Ahmad Khan[4], Abdulfatah M. Alayoubi[2], Morad Qarmali[2], Muhammad Latif [2, 5, *]

[1]Department of Biology, Allama Iqbal Open University, Islamabad, Pakistan

[2]Department of Basic Medical Sciences, College of Medicine, Taibah University, Madinah, 42353, Saudi Arabia

[3]Federal Medical College, Islamabad, Pakistan

[4]Gomal Centre of Biochemistry and Biotechnology, Gomal University, Dera Ismail Khan (D.I. Khan) 29050, Khyber Pakhtunkhwa, Pakistan

[5]Center for Genetics and Inherited Diseases, Taibah University, Madinah 42353, Saudi Arabia

***Correspondence:** Rizwana Kousar (rizwana.kousar@aiou.edu.pk), Muzammil Ahmad Khan (m.ahmad@gu.edu.pk), Muhammad Latif (mmuradkhan@taibahu.edu.sa)

Table S1. Prediction of structural stability and evolutionary conservation for selected deleterious SNPs in *MCPH1*.

	**1-Mutant**				**Consurf**		
**S.NO.**	**Amino acid Change**	**SVM2 Prediction Effect (Stability)**	**DDG Value Prediction Kcal/mol**		**ConSurf conservation Score**	**Functional/structural**	**Exposed/Buried**
1	**A11D**	**Decrease**	**-0.68**		**9/Conserved**	**Structural residue**	**Buried residue**
2	**H49D**	**Decrease**	**-0.86**		**9/Conserved**	**Functional residue**	**Exposed residue**
3	**T59I**	**Decrease**	**-0.38**		**9/Conserved**	**Functional residue**	**Exposed residue**
4	**W60S**	**Decrease**	**-0.02**		**9/Conserved**	**Structural residue**	**Buried residue**
5	**W60C**	**Decrease**	**-1.5**		**9/Conserved**	**Structural residue**	**Buried residue**
6	**L74P**	**Decrease**	**-1.43**		**9/Conserved**	**Structural residue**	**Buried residue**
7	**C79G**	**Decrease**	**-1.18**		**9/Conserved**	**Structural residue**	**Buried residue**
8	C110Y	Increase	0.76		7/Conserved	Structural residue	
9	C110R	Decrease	-0.21		7/Conserved	Structural residue	
10	**R693C**	**Decrease**	**-0.92**		**9/Conserved**	**Functional residue**	**Exposed residue**
11	**R703C**	**Decrease**	**-1.02**		**9/Conserved**	**Functional residue**	**Buried residue**
12	**C705Y**	**Decrease**	**-0.34**		**9/Conserved**	**Structural residue**	**Buried residue**
13	**W706S**	**Decrease**	**-1.46**		**9/Conserved**	**Structural residue**	**Buried residue**
14	Y710C	Decrease	-1		8/Conserved		Buried residue
15	W712G	Decrease	-1.82		7/Conserved		Buried residue
16	W712S	Decrease	-1.4		7/Conserved		Buried residue
17	W712C	Decrease	-1.45		7/Conserved		Buried residue
18	**G720V**	**Decrease**	**-0.29**		**9/Conserved**	**Functional residue**	**Exposed residue**
19	**L755P**	**Decrease**	**-1.86**		**9/Conserved**	**Structural residue**	**Buried residue**
20	**L781P**	**Decrease**	**-1.38**		**9/Conserved**	**Structural residue**	**Buried residue**
21	G783R	Decrease	-0.36		Average		Exposed residue
22	A793D	Increase	0.69		9/Conserved	Structural residue	Buried residue
23	**D818H**	**Decrease**	**-0.41**		**9/Conserved**	**Functional residue**	**Exposed residue**

Table S2. A summary of available ClinVar annotations for the prioritized missense variants.

S.No.	SNP ID	Structure	gnomAD	ClinVAR
1	rs767448816	A11D mutant	0.000008061	Not Reported
2	rs372088330	H49D mutant	0.000003718	Uncertain Significance
3	rs1333885096	T59I mutant	0.000002479	Not Reported
4	rs1240826704	W60C mutant	0.000001240	Not Reported
5	rs751733149	W60S mutant	0.000004338	Not Reported
6	rs1278624882	L74P mutant	0.000001862	Uncertain Significance
7	rs1349038447	C79G mutant	6.203e-7	Not Reported
8	rs1208842915	L781P mutant	6.195e-7	Not Reported

Table S3. Solvent accessibility and Stability profile of *MCPH1*.

S.No.	Amino acid Change	Class Assignment	Relative surface Accessibility (RSA) %	Absolute surface Accessibility (ASA) Ao	Secondary structure
1	**A11D**	Buried	0	1	Strand
2	**H49D**	Buried	6	10	Coil
3	**T59I**	Buried	21	29	Helix
4	**W60S**	Buried	14	34	Helix
5	**W60C**	Buried	14	34	Helix
6	**L74P**	Exposed	36	67	Helix
7	**C79G**	Buried	2	3	Helix
8	**L781P**	Not given	27	55	Helix

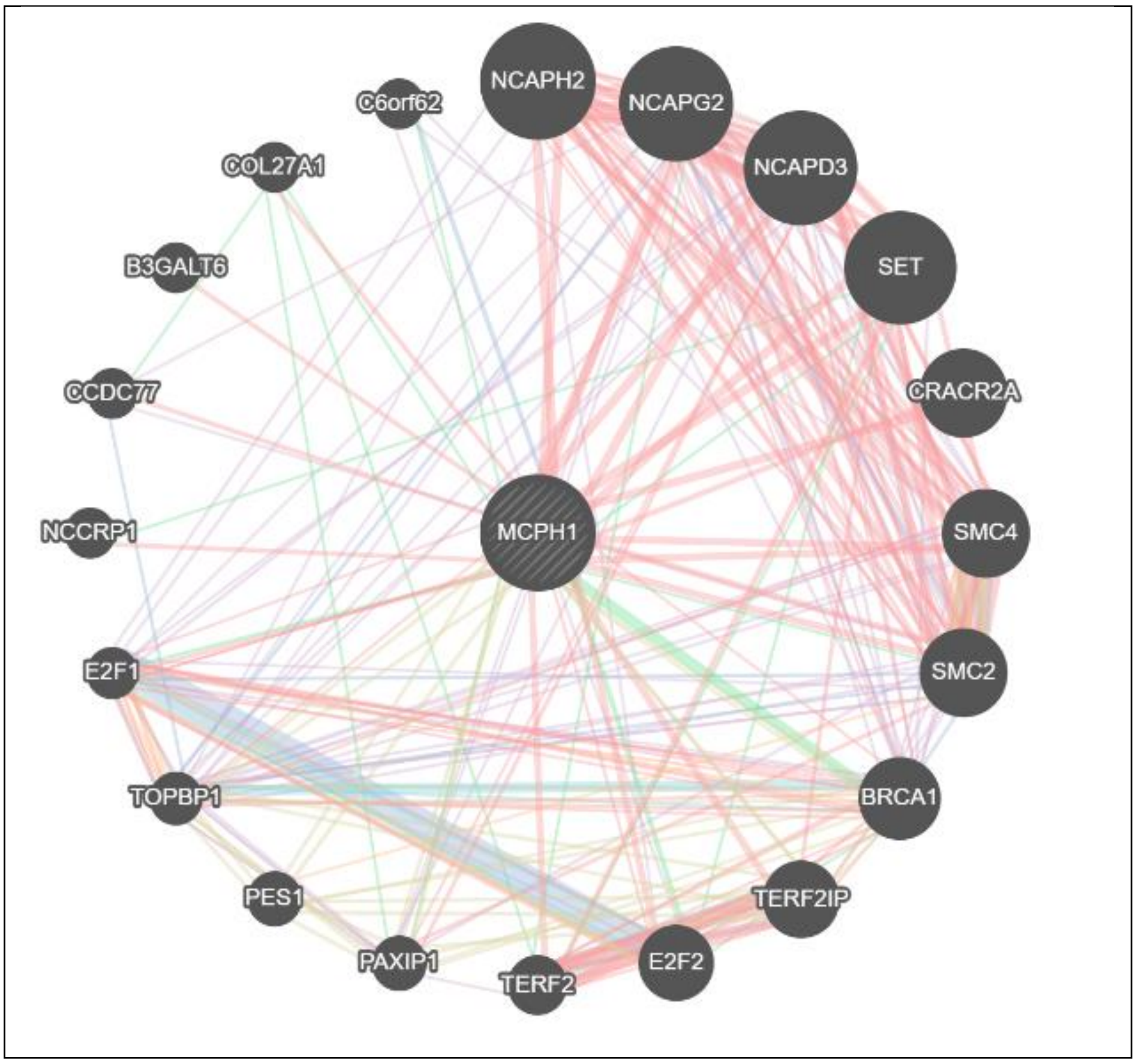

(a). GeneMANIA prediction

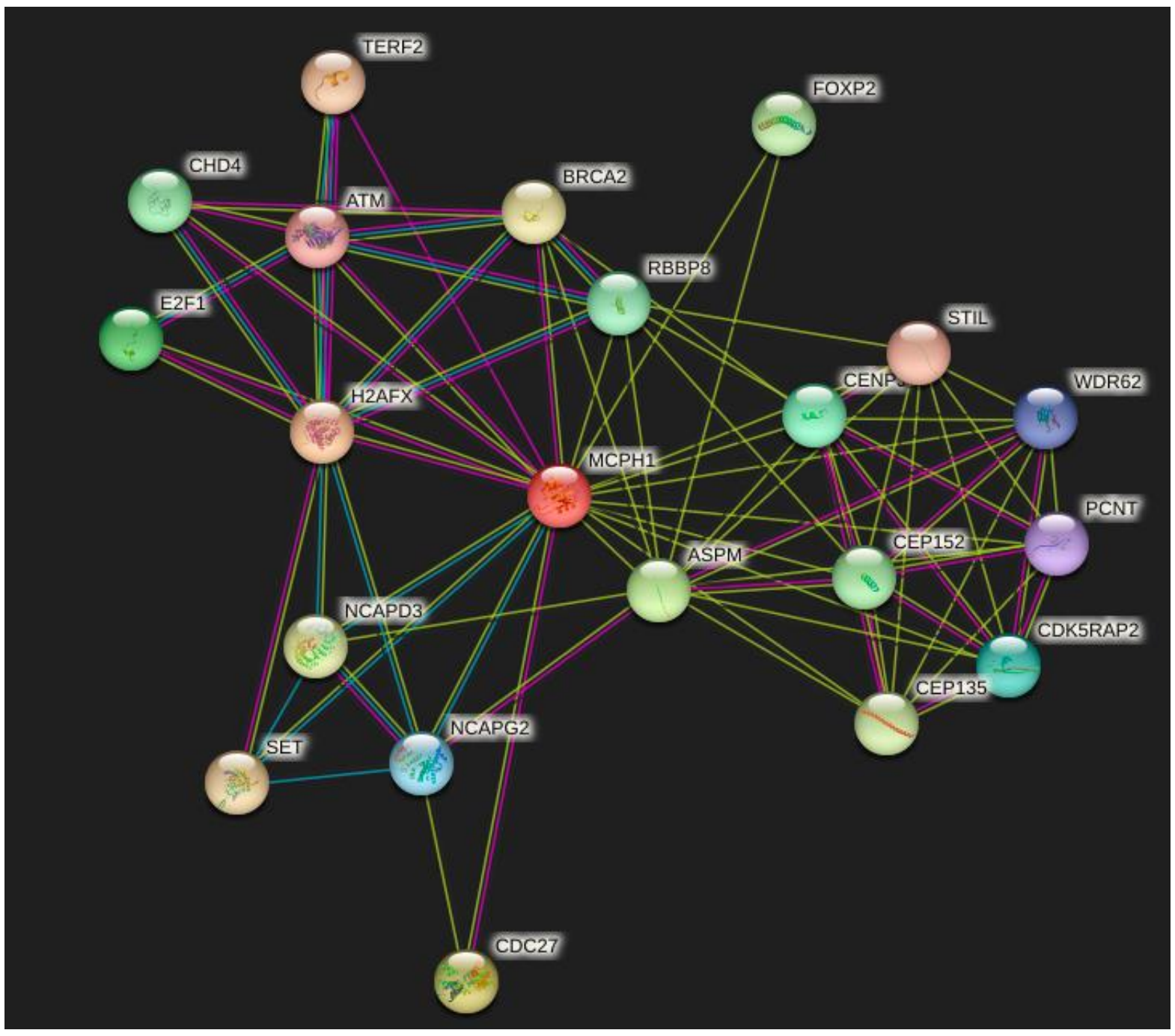

(b). String prediction

Figure S1. The interaction network of *MCPH1* with other genes: (**a**) GeneMANIA prediction, (**b**) String prediction
